# The rim sign: FDG-PET/CT pattern of pulmonary infarction

**DOI:** 10.1007/s13244-012-0189-5

**Published:** 2012-08-18

**Authors:** Michael Soussan, Edmond Rust, Gabriel Pop, Jean-François Morère, Pierre-Yves Brillet, Véronique Eder

**Affiliations:** 1 AP-HP, Department of Nuclear Medicine, Avicenne hospital, University Paris 13, Bobigny, France; 2 Department of Nuclear Medicine, CHU Hautepierre, Strasbourg, France; 3 AP-HP, Department of Oncology, Avicenne hospital, University Paris 13, Bobigny, France; 4 AP-HP, Department of Radiology, Avicenne hospital, University Paris 13, Bobigny, France; 5 AP-HP, Department of Nuclear Medicine, Avicenne hospital, 125 rue de Stalingrad., 93000 Bobigny, France

**Keywords:** Pulmonary infarction, Pulmonary embolism, FDG-PET/CT, Pattern

## Abstract

**Objective:**

We aimed to describe a pattern of rim uptake observed in lung infarction on FDG-PET/CT, called the “rim sign.” It was defined as a continuous slight FDG uptake along the border of a subpleural consolidation without uptake within the consolidation.

**Methods:**

We retrospectively reviewed the FDG-PET/CT studies of 400 patients referred for thoracic oncological workup from November 2010 to July 2011. The rim sign was observed in six patients who had confirmed pulmonary infarction (PI) on MDCT showing acute pulmonary embolism (*n* = 4) or tumoral arterial obstruction (*n* = 2).

**Results:**

Eight PIs in the six patients exhibited the rim sign with slight uptake (median SUV_max_: 3.6, 2.2–6.8) and median size of 48.5 mm (30–74). On MDCT, central lucencies, triangular shape and vessel sign were observed in 5/8, 4/8 and 1/8 cases, respectively. Two out of the eight PIs exhibited only the rim sign and none the suggestive MDCT sign.

**Conclusion:**

The rim sign is easily recognisable at FDG-PET/CT and is strongly suggestive of PI. This pattern can be observed even in the absence of suggestive findings on MDCT. Recognition of this sign should prompt investigations for pulmonary embolism.

***Main Messages*:**

• *The rim sign is a slight FDG uptake around an area of subpleural consolidation*

• *The rim sign is strongly suggestive of pulmonary infarction*

• *Recognition of the rim sign should prompt investigations for pulmonary embolism*

## Introduction

Incidental pulmonary embolism is a not an uncommon diagnosis in patients with cancer. There is a wide body of evidence showing the higher incidence of deep venous thrombosis and pulmonary embolism in this population, for both in- and outpatients, supporting the existence of a link between cancer and activation of the hemostatic system [1, 2]. Anticancer therapy is also recognised to increase the risk of deep venous thrombosis and pulmonary embolism [2]. Positron emission tomography/computed tomography with fluorodeoxyglucose (FDG-PET/CT) being now a standard procedure in the management of several malignancies, most cancer patients undergoing FDG-PET/CT are at risk for pulmonary embolism. Pulmonary embolism is associated with pulmonary infarction in about 25 % of cases, often appearing as a non-specific peripheral wedge-shaped parenchymal consolidation [3, 4]. As most of FDG-PET/CT protocols do not include contrast-enhanced CT, pulmonary infarction might appear as a unique consolidation with variable FDG uptake that can mimic a primary tumor or lead to an overestimation of the tumor burden in patients with various malignancies [5]. That the quantitative parameters offered by PET technology are hampered in making the distinction between infarction and tumoral uptake because of the inflammatory processes associated with pulmonary infarction is noteworthy. Thus, occult lung infarction may induce false interpretation of FDG-PET/CT in the staging of pulmonary malignancies because of the wide overlap between the maximum standardised uptake values (SUV_max_) measured in pulmonary infarction and in tumoral lesions [5].

We present the FDG-PET/CT investigations of six patients with confirmed pulmonary infarction and suggest a distinguishable metabolic pattern, the rim sign, which may help the nuclear medicine physician make this difficult diagnosis in daily practice.

## Materials and methods

This study was institutional review board approved, with waiver of informed consent. We retrospectively reviewed consecutive FDG-PET/CT studies of 400 patients referred for thoracic oncological workup from November 2010 to July 2011. The rim sign was observed in six patients (5 men and 1 woman; age 53–89, median 56) who were included in this case series study. The rim sign was defined on FDG-PET/CT as the association of (1) a continuous slight FDG uptake along the border of a subpleural lung consolidation and (2) the absence of FDG uptake within the consolidation. Patients were referred for lung cancer initial staging (*n* = 3), therapy response assessment (*n* = 1) and characterisation of suspicious lung lesions (*n* = 2). Pulmonary infarction was confirmed in all patients on contrast-enhanced MDCT and patient follow-up (clinical examination and chest x-ray) for at least 6 months after FDG-PET/CT. Diagnostic criteria for pulmonary infarction was either a peripheral consolidation with adjacent subsegmental PE on contrast-enhanced MDCT [3] (*n* = 4) or proximal tumoral arterial obstruction in the segment of the pulmonary infarction (*n* = 2) with exclusion of another cause of peripheral consolidation such as obstructive pneumonia or lung tumor. Obstructive pneumonia with central abcedation was excluded by the visualisation of normally aerated lung parenchyma between the proximal tumoral obstruction and the subpleural consolidation. Contrast-enhanced MDCT was either performed in the same session as the PET/CT examination or on the following day.

All FDG-PET/CT images were obtained using 16-slice PET/CT (Gemini TF, Philips Medical Systems, The Netherlands) 60 min following intravenous injection of 3 MBq/kg FDG. The field of view was from the base of the skull to mid-thigh. CT images were obtained with or without contrast media injection using the following parameters: 120 KV, 100 mAs, collimation 16 × 1.5 mm, pitch of 0.69, slice thickness: 3 mm and increment of 1.5 mm. CT data were used for the attenuation correction. All patients had a serum glucose level <7.7 mmol/l at the time of injection. On PET images, semiquantitative analysis with measurement of the maximal standardised uptake value (SUV_max_) of the lesion exhibiting the rim sign was performed. On MDCT images, morphological characteristics associated with pulmonary infarction were recorded: triangular shape, vessel sign and central lucencies [3]. Triangular shape corresponds to a consolidation with an apex in a more central portion of the lung than the broad base. The vessel sign defines the presence of an enlarged vessel leading to the apex of a consolidation. Central lucencies were defined as round foci of hypoattenuation in the central portion of a consolidation.

## Results

Patients’ characteristics including clinical context, mechanism, topography, size, SUV_max_ and morphologic characteristics of pulmonary infarctions are reported in Table 1.

**Table 1:** Patients’ characteristics and pulmonary infarction imaging findings

	Sex	Age (years)	Clinical context	Primary tumor: histopathological type, localization and SUVmax	Pulmonary infarction characteristics						
					Mechanism	Topography	Size (mm)	SUVmax	Triangular shape	Vessel sign	Central lucencies
1	M	55	Lung cancer initial staging	Lung Adenocarcinoma; RLL; SUVmax: 21.8	PE	RLL	34	3,7	+	−	+
						RUL	50	4,6	−	+	−
2	M	53	Lung cancer initial staging	Small cell lung cancer; mediastinum; SUVmax: 11.7	Tumoral obstruction	LUL	60	4,8	+	−	+
						LUL	30	3,5	+	−	+
3	F	89	Lung cancer initial staging	Squamous cell lung cancer; LUL; SUVmax: 9.5	PE	RUL	47	2,7	+	−	+
4	M	63	Suspicion of lung cancer	*	PE	RLL	60	3,1	−	−	−
5	M	57	Suspicion of lung cancer	*	PE	ML	33	2,2	−	−	−
6	M	53	Therapy response assessment	Squamous cell lung cancer: ML; SUVmax: 9.5	Tumoral obstruction	RLL	74	6,8	−	−	+

*PE* pulmonary embolism; *RLL* right lower lobe; *LLL* left lower lobe; *RUL* right upper lobe; *LUL* left upper lobe; *ML* middle lobe

*These patients were suspected to have lung cancer because of a solitary pulmonary mass but were eventually diagnosed as having a pulmonary infarction without underlying tumoral disease

**Fig. 1:**
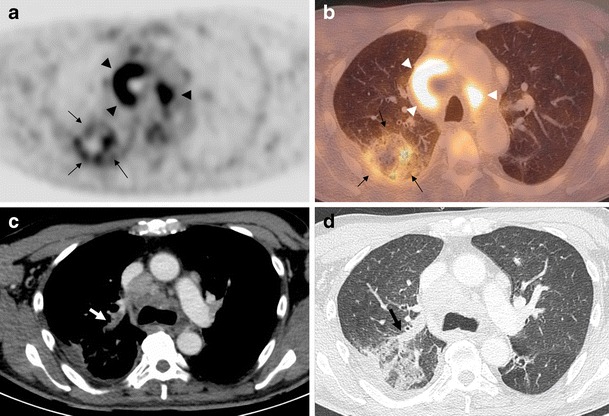
A 55-year-old man with a metastatic right lower lobe lung adenocarcinoma (patient 1). FDG-PET/CT images showed a right upper lobe subpleural consolidation exhibiting the rim sign (**a**, axial PET; **b**, axial fusion, *arrows*). Metastatic mediastinal adenopathies were clearly visible (*arrowheads*). Combined enhanced MDCT confirmed the acute pulmonary embolism (**c**, *arrow*) and showed the vessel sign associated with the right pulmonary infarction (**d**, *arrow*)

**Fig. 2:**
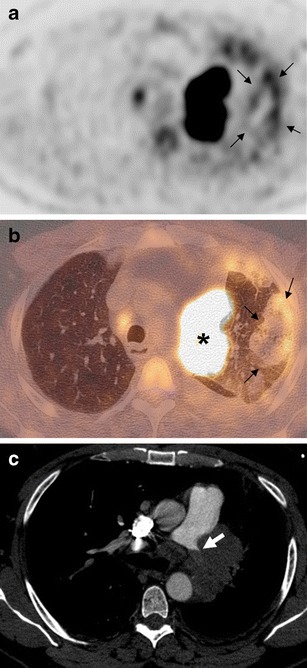
A 53-year-old man referred for small cell lung cancer staging (patient 2). FDG-PET/CT images showed a left upper lobe subpleural consolidation with triangular shape and central lucencies, exhibiting the rim sign (**a**, axial PET; **b**, axial fusion, *arrows*). Mediastinal tumor with high FDG uptake is clearly visible (**b**, *star*). MDCT showed complete arterial obstruction of the left pulmonary artery (**c**, *arrow*)

**Fig. 3:**
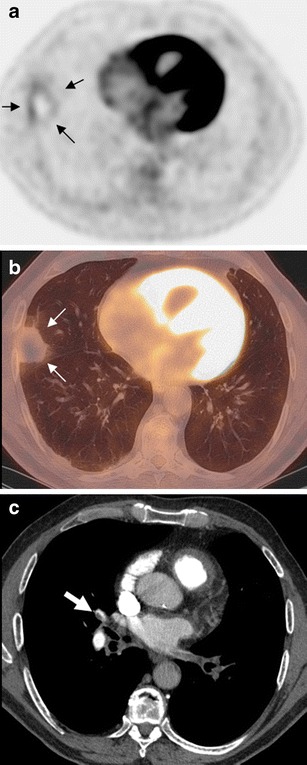
A 57-year-old man referred for workup of a solitary pulmonary mass (patient 5). FDG-PET/CT images showed a right subpleural consolidation with the rim sign (**a**, axial PET; **b**, axial fusion, *arrow*). MDCT confirmed acute pulmonary embolism (**c**, *arrow*) showing an adjacent subsegmental pulmonary embolism. Note the absence of any suggestive signs of pulmonary infarction on MDCT. Patient follow-up confirmed the absence of associated lung tumor

Eight pulmonary infarctions were diagnosed in the six patients. Five pulmonary infarctions were related to acute pulmonary thromboembolism (Fig. 1), and three pulmonary infarctions were caused by hilar tumoral arterial obstruction (Fig. 2). Median size of the pulmonary infarctions was 48.5 mm (30–74 mm). Central lucencies and triangular shape were observed in 5/8 and 4/8 cases, respectively. The vessel sign was present in only one lesion. Two out of the eight pulmonary infarctions appeared as a solitary pulmonary mass and only exhibited the rim sign, without any of the suggestive MDCT sign (Fig. 3).

FDG uptake was considered as slight in all cases. Only small parts of the FDG rim uptake presented a marked intensity, with the measured SUV_max_ ranging between 2.2 and 6.8 (median 3.6). In all cases with PE, no FDG uptake was observed in the arterial thrombus.

## Discussion

As recently pointed out by Hofman and Hicks [6], pattern recognition appears to be of great value in the field of PET imaging. Hence, nuclear medicine physicians are already used to identify some benign conditions based on the distribution of FDG uptake, such as brown fat activation and symmetric hilar node uptake. Beyond all the useful quantitative parameters offered by PET technology, the next step might be the definition of distinguishable metabolic patterns. This approach has already proved its usefulness in the response assessment of several cancers, where the inflammatory processes associated with therapy hamper a quantitative evaluation of tumoral uptake changes [7]. FDG uptake intensity is useless in making the distinction among inflammatory, infectious and tumoral lung lesions [8]. Indeed, post-stenotic atelectasis, granulomatous lung disease and many infectious lesions have demonstrated various FDG uptake patterns and can therefore mimic cancer [9]. Regarding pulmonary infarction, there is an overlap of the SUV_max_ values with lung tumors [5].

In this study we aimed to point out a distinctive pattern of pulmonary infarction called the rim sign. This metabolic pattern is consistent with pathologic data on pulmonary infarction, showing that consolidation in pulmonary infarction is mainly caused by central blood alveolar filling with a peripheral inflammatory reaction including a foamy macrophage margin at the edge of central necrosis [10]. FDG uptake is certainly related to this inflammatory peripheral process. A rim of FDG uptake in a pulmonary infarction was described in one patient by Badr [11].

Such a metabolic pattern is however not specific for pulmonary infarction. Hence, several consolidations such as lung tumors with central necrosis or lung abscess can show intense peripheral FDG uptake with low central FDG uptake. Nevertheless, in this study, the eight pulmonary infarctions exhibited a slight, thin and continuous FDG uptake in all cases, with a median SUV_max_ value of 3.6, which is close to those reported previously [5]. Tumors with central necrosis or lung abscess typically appear as round masses with stronger and thicker FDG uptake [12, 13].

The association of the rim sign with one or more of the morphologic characteristics associated with pulmonary infarction may increase diagnostic confidence. Among them, central lucencies and the vessel sign are reported as the most specific CT findings (specificity 98 % and 89 %, respectively) [3]. Unfortunately, all of these CT findings were reported to have a very low sensitivity. Interestingly, in two patients, the subpleural consolidation exhibited none of the MDCT signs suggestive of pulmonary infarction. This means that the rim sign could be the only imaging criterion to suggest a pulmonary infarction in such cases. It is noteworthy that in all cases of this series pulmonary infarctions were detected incidentally. Patients did not report symptoms suggestive of acute PE, such as acute chest pain or dyspnea. Besides, arterial thrombi can be associated with FDG uptake and thus help to suggest pulmonary embolism [5], but this was not observed in our study.

Consideration of the patient’s clinical data and previous imaging results is an additional way to avoid diagnostic pitfalls. Thus, a parenchymal consolidation with the rim sign appearing aside a known or under-treatment lung tumor should raise the question of pulmonary infarction as a differential diagnosis.

This retrospective case series study has limits. First, the design was retrospective and the number of patients included small. Second, this case series study did not allow an evaluation of the sensitivity and specificity of the rim sign, and a prospective study is needed to determine its diagnostic accuracy.

In conclusion, the rim sign, defined as a slight and continuous FDG uptake at the border of a peripheral lung consolidation, is easily recognisable at FDG-PET/CT and is strongly suggestive of pulmonary infarction. This pattern can be observed even in the absence of any suggestive finding of pulmonary infarction on MDCT. In daily practice, this pattern may help the physician to refer patients for lung perfusion scintigraphy or contrast-enhanced MDCT for pulmonary embolism assessment.
